# The Nature of Progression in Parkinson’s Disease: An Application of Non-Linear, Multivariate, Longitudinal Random Effects Modelling

**DOI:** 10.1371/journal.pone.0076595

**Published:** 2013-10-18

**Authors:** Lisa Kuramoto, Jacquelyn Cragg, Ramachandiran Nandhagopal, Edwin Mak, Vesna Sossi, Raul de la Fuente-Fernández, A. Jon Stoessl, Michael Schulzer

**Affiliations:** 1 Centre for Clinical Epidemiology & Evaluation, Vancouver Coastal Health Research Institute, Vancouver, British Columbia, Canada; 2 Pacific Parkinson’s Research Centre, The University of British Columbia, Vancouver, British Columbia, Canada; 3 Department of Physics & Astronomy, The University of British Columbia, Vancouver, British Columbia, Canada; The University of Chicago, United States of America

## Abstract

**Background:**

To date, statistical methods that take into account fully the non-linear, longitudinal and multivariate aspects of clinical data have not been applied to the study of progression in Parkinson’s disease (PD). In this paper, we demonstrate the usefulness of such methodology for studying the temporal and spatial aspects of the progression of PD. Extending this methodology further, we also explore the presymptomatic course of this disease.

**Methods:**

Longitudinal Positron Emission Tomography (PET) measurements were collected on 78 PD patients, from 4 subregions on each side of the brain, using 3 different radiotracers. Non-linear, multivariate, longitudinal random effects modelling was applied to analyze and interpret these data.

**Results:**

The data showed a non-linear decline in PET measurements, which we modelled successfully by an exponential function depending on two patient-related covariates duration since symptom onset and age at symptom onset. We found that the degree of damage was significantly greater in the posterior putamen than in the anterior putamen throughout the disease. We also found that over the course of the illness, the difference between the less affected and more affected sides of the brain decreased in the anterior putamen. Younger patients had significantly poorer measurements than older patients at the time of symptom onset suggesting more effective compensatory mechanisms delaying the onset of symptoms. Cautious extrapolation showed that disease onset had occurred some 8 to 17 years prior to symptom onset.

**Conclusions:**

Our model provides important biological insights into the pathogenesis of PD, as well as its preclinical aspects. Our methodology can be applied widely to study many other chronic progressive diseases.

## Introduction

Parkinson’s disease (PD) is one of the commonest neurodegenerative disorders, with a median incidence of 160 per 100,000 (range 62 to 332 per 100,000) in the American population aged 65 years and above [1]. The burden of the illness is quite substantial in the aging population with an estimated cost of approximately $2.5–5 billion annually [2], [3]. The cardinal manifestations of PD include motor symptoms such as tremor, rigidity, slowness (bradykinesia) or poverty of movements (hypokinesia) affecting daily activities. These cardinal symptoms are due to neuronal loss and degeneration in the nigrostriatal dopamine projection in the brain and consequent loss of striatal dopamine content. Over the course of the disease, widespread neuronal loss leads to postural instability with attendant falls, mood problems (which may also antedate the onset of motor symptoms), cognitive impairment, and autonomic disturbances, further diminishing the quality of life. Established treatment options for PD are by and large symptomatic and have little influence in altering the underlying neuropathological process. In this context, longitudinal study of disease progression has relevance not only for better understanding the natural history of the disease but also for specifically improving the design and timing of potential neuroprotective interventions when they become available. However, assessment of progression using only clinical measures and motor scores is fraught with the potential subjective confound of prolonged symptomatic benefits resulting from the therapeutic agents [4]. Hence, objective assessment of progression using radiotracer imaging such as Positron Emission Tomography (PET) with suitable dopaminergic drug washout has been increasingly utilized in recent years [5], [6]. An additional advantage of this functional imaging is the possibility of quantification of alterations in the metabolic or neurotransmitter function or level of denervation (neuronal loss) with reference to control values.

We have previously used multitracer PET to understand the compensatory changes in the early stages of the disease and in asymptomatic mutation carriers of genetic forms of PD [7], [8]. These PET scans assess presynaptic dopaminergic integrity using: 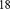
F-6-fluoro-L-dopa (FD) reflecting the synthesis of dopamine (DA) from levodopa and its storage in synaptic vesicles, 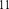
C-(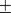)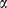-dihydrotetrabenazine (DTBZ) labeling vesicular monoamine transporter type 2 (VMAT2), responsible for the packaging of monoamine neurotransmitters into synaptic vesicles and estimating DA terminal density, and 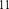
C-d- *threo*-methylphenidate (MP) labeling the membrane DA transporter (DAT), responsible for reuptake of DA from the synapse into the neurons, thereby terminating its action at DA receptors. The quantitative measures include regional values of uptake constant (K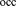) using FD PET and binding potential (proportional to the maximum specific binding [B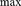] divided by the equilibrium dissociation constant K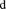) using DTBZ PET and MP PET with reference tissue (occipital cortex) input function. These measures reflect the affinity for and occupancy by these radiotracers for the metabolic substrates or transporters involved in central dopamine processing (for more detailed information see [5], [9]). Serial scanning using these radiotracers helps to capture the progressive changes in neuronal density or striatal dopamine processing and thus provides an objective measure of the neurochemical and neurobiological progression of the disease. The temporal change in the serial measure(s) aids in charting the trajectory of the neurobiological alterations *in vivo* and serves as a biomarker in following disease progression. As the dopaminergic dysfunction in sporadic PD shows an anteroposterior gradient among the striatal subregions and lateral asymmetry (manifesting as the more and less affected sides) at least in the initial stage of the disease, comparing the trajectories within and between striata provides useful information on the spatial dynamics of disease progression. Such information aids in better understanding the mechanisms underlying aetiopathogenesis.

**Figure 1:**
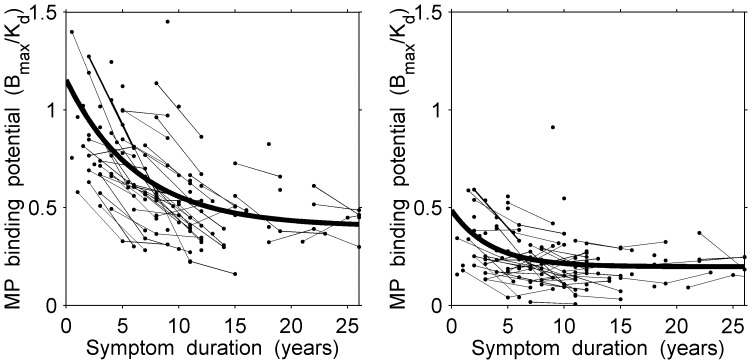
Scatter plot of observed longitudinal responses (MP measurements) in the less affected side for anterior putamen (left panel) and posterior putamen (right panel) with the superimposed non-linear curves derived from the random effects model for patients with an average age at symptom onset of 53 years.

Multivariate longitudinal data are characterized by multiple responses measured at multiple times for each subject [10]. Our multivariate PD PET data consist of simultaneous measurements taken from several regions of the brain. At each of 3 successive visits, 4 years apart, data were collected for each patient in four regions of the brain: the anterior, mid, and posterior putamen, and the caudate, from both the less affected and the more affected sides. Thus, a combination of 3 PET tracers, 4 regions, and 2 sides of the brain resulted in a total of 24 simultaneous measurements per patient per visit. However, the measurements were observed at different disease stages for each patient. With the increasing duration of the symptomatic disease, the measurements were typically seen to decrease exponentially over time, usually levelling off at an asymptotic level (Figure 1).

A variety of statistical methods have been applied in the past to study disease progression in PD. These methods did not allow simultaneously for both temporal and spatial inferences [11]–[17]. Authors have developed models which addressed at least one, but not all, of the multivariate, longitudinal, or non-linear aspects of the data [18]–[21]. Recently, Marshall generalized the non-linear random effects model to allow for multivariate responses and for missing data [10]. Our new model handles multivariate responses and longitudinal data, allows for the number and timing of observations to differ across individuals, allows for the comparisons of correlated parameters, and has increased power.

We have applied Marshall’s non-linear random effects model for multivariate measurements to analyze our PD data. In the Methods and Results, we demonstrate the usefulness of this statistical method, which has allowed us to characterize the temporal and spatial aspects of the progression of the disease, and to explore the pre-symptomatic phase of PD. The Discussion provides a summary of our findings.

## Methods and Results

### 2.1 Ethics Statement

All patients gave written informed consent and the study was approved by the Clinical Research Ethics Board of the University of British Columbia.

### 2.2 Data

Of the 78 PD patients included in the study, the total number receiving scans for the tracers on the first, second, and third visit were 78, 57 and 21, respectively. The average age at symptom onset was 53 years (SD: 10 years). To illustrate the regional and temporal correlations we encountered in the data, the median correlation in MP measurements between the anterior and posterior putaminal regions was 0.69; the overall median correlation across successive time points was 0.74.

In addition, a total of 35 normal subjects provided corresponding longitudinal control scans for each of the three tracers, from the same regions for the left and right sides of the brain. The total number of normal subjects with scans at the first, second, and third visit were 35, 29, and 18, respectively. These serial measurements were also taken 4 years apart. The average age of the normal control subjects at their first observation was 55 years (SD: 15 years). For the normal subjects, tracer measurements were modelled as a *linear* function of age at the time of visit with a random effect for the intercept (see model (8)). The longitudinally derived linear regressions for all three tracer measurements provided a good fit to the data. Non-linear models, both polynomial and exponential, were tested for their goodness-of-fit in each case, but failed to provide significant improvement to the linear fit.

All the data were acquired on the same scanner, the Siemens ECAT 953B [22], using identical scanning protocols; care was taken to ensure that data quantification accuracy and reproducibility were not affected by any instrumentation software upgrade. All measurements for the PD patients and for the normal subjects were obtained by the same PET analyst.

### 2.3 Model

Let 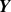 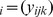 be an 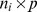 response matrix taken on patient 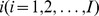 at occasion 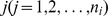 on response 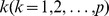, where response may refer to region, side, or tracer. Thus,


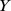
 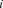 = 
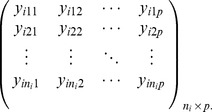


Let 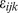 be the error term associated with 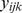. The non-linear random effects model is given by
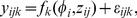
(1)where 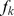 is a non-linear function of the parameter vector 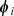 and covariates 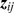 [10]. The parameter vector 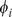 consists of two components: a vector of fixed population effects, 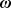 (defined below), and a vector of patient-related random effects, 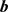 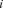. The random effects, 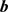 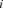, are assumed to follow a multivariate normal distribution.

Based on visual, biological and statistical evidence, the best fit for the observed decline in our measurements in PD patients with increasing symptom duration was found to be an exponential function (Figure 1) depending on two patient-related covariates: the duration 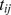 since symptom onset, and the age 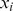 at symptom onset; such that, 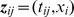.

Thus, the proposed mean structure for the 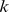
th response for patient 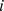 at visit 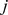 is
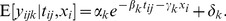
(2)


Our model (2) is a special case of the more general model (1). Each of the four parameters, 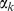, 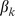, 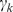, and 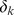, can be associated with a random effect. We let 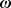 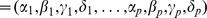 be the parameter vector and 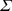 the corresponding covariance matrix of their estimates. Further details may be found in [10].

Independence was assumed among patients, and error terms were assumed to be uncorrelated with the random effects. The parameter estimates for this model were derived using the EM algorithm, which was extended to handle estimation of models with random effects and missing data [10]. In these models, missing data are assumed to be missing at random (see also Discussion).

Modelling multivariate data directly permits comparisons of correlated parameters, parametric functions and the resulting curves. The progression of PD over time has long been suspected to be non-linear in nature [11]. The precise estimation of the coefficient of curvature 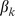 in model (2) above for each of the response variables is of prime importance in capturing this potential non-linearity in the evolution of the disease. Longitudinal data offer a far more precise estimate of this coefficient than do cross-sectional data (see Section 2.11).

Clinical evidence suggests that patients who developed PD symptoms at a younger age may have a different time course of their disease from those whose symptoms began at a later age. The coefficient 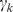 in model (2) addresses the effect of age at symptom onset on the corresponding pattern of disease progression.

The asymptote 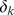 in our model refers to the final level of damage in each response measurement 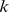 when the disease progression, as observed by the PET tracers, appears to level off and beyond which no further substantial decline is seen. We interpret 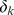 as the final level of damage: it represents the mean PET measure as time since symptom onset, 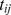, approaches infinity. The comparison of asymptotes between regions, or between sides, indicates whether regions or sides that may have started out as affected to different degrees, progress to have a similar final level of involvement or remain distinct. For PD, the estimated value of each curve at the time of symptom onset (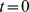) identifies the degree of damage to the dopamine system when symptoms begin to manifest themselves. From a clinical perspective, for a patient at age 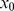, significant losses in PD tracer curve intercepts, as represented by 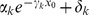 (when 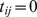 and 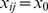 in model (2)), relative to the corresponding age-matched normal controls, as represented by 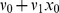 (when 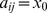 in model (8)), suggest that nerve terminals have been lost prior to the onset of symptoms. Once nerve terminals are lost, a difference in rates of decline between tracers 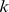 and 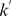, 
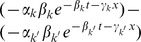
, may be due to compensatory changes or to differential involvement of various regions (see Section 2.10).

The overall rate of decline at any time is affected by all three parameters 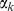, 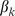, and 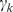. We limited our model to the well established factors of age of onset of symptoms and duration of symptoms; various other initiating factors are suspected in PD but were not included in the model. These suspected, initiating factors may have potentially resulted in differential involvement at different regions.

The time of *disease* onset (i.e., the time of initial damage to the dopaminergic function) is known to precede the time of *symptom* onset. Back-estimation of the progression curve into the preclinical and presymptomatic duration of disease in each PET measurement was carried out, and its time of intersection with the corresponding normal control line was estimated, to give an indication of how long prior to symptom onset the activity of the disease might have begun (see Section 2.9). This helped to examine possible causative factors, at that initial time point, and shed some new light on the mechanism of disease onset.

We have selected a variety of examples below in order to illustrate the diversity of statistical methods that were applied to different combinations of tracers and regions, and the great deal that we were thus able to learn about the many aspects of the progression of PD. In Sections 2.4 to 2.6 we illustrate our methods with examples of joint estimation of a pair of regions, specifically comparing parameter values, intercepts and rates between these regions. In Section 2.7 we further show the estimation of potential convergence, divergence, or parallelism between the less affected and the more affected sides for specific regions. In Section 2.8, the effect of the age of onset of symptoms is related to the degree of nerve terminal loss at 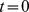. Estimation of the preclinical duration of disease is illustrated in Section 2.9. Finally, in Section 2.10, we compare features of the curves of different (standardized) tracers.

We used SAS 9.1 software (SAS System version 9.1 for Windows, SAS Institute Incorporation, Cary, NC, USA, 2002–2003) and R software (R version 2.3.0 for Windows, R Foundation for Statistical Computing, Vienna, Austria, 2006) for all statistical analyses. The SAS Procedure NLMIXED was used to estimate parameter values. R was used to estimate and test non-linear functions of the parameters using the delta method.

### 2.4 Estimation and Comparison of Parameter Values: Anterior vs Posterior Putamen Remain Functionally Different Over the Course of the Illness

We compared parameter estimates across regions by letting the 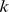
th response in our model represent the 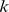
th region. Thus, as an example, let 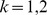 represent, respectively, the anterior and posterior putamen. Estimates of the eight parameters, 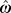 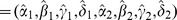, and of their covariance matrix, 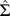, are readily available from the fitted model. The variance of the difference in parameter estimates between regions 1 and 2 can be approximated based on 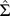. For example, the variance of the difference between the asymptotes is approximated by
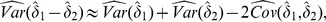
(3)and an approximate 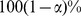 confidence interval [CI] for the difference between the 2 asymptotes is given by
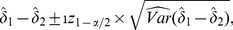
(4)where 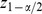 is the 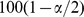
th percentile of the standard normal distribution. Similarly, we estimate the differences between 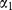 and 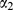, 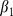 and 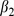, and 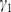 and 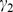.

To illustrate, we compared parameter estimates for the MP tracer between the anterior and posterior regions of the putamen in the less affected side. Figure 1 shows the longitudinal scatter plots of the data together with the superimposed fitted curves for our patients, whose average age at symptom onset was 53 years. Visually, measurements in the anterior putamen appear to be higher than in the posterior putamen.

Table 1 shows the corresponding estimated parameter values for the anterior putamen (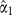, 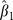, 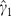, 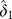) and for the posterior putamen (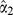, 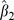, 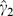, 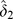). Several parameter estimates were statistically significant at the 5% level. Significant curvature was detected in both the anterior putamen (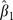 = 0.16, 95% CI 0.10–0.22) and posterior putamen (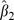 = 0.29, 95% CI 0.11–0.46). Comparisons between parameter estimates showed that the asymptote in the anterior putamen was higher by 0.20 B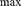/K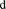 than in posterior putamen (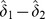 = 0.20, 95% CI 0.12–0.29). From this result, clinicians concluded that these two regions remain functionally different throughout the course of the illness.

**Table 1:** Estimated nonlinear random effects model of responses (MP measurements) in the less affected side for anterior putamen and posterior putamen.

Parameter	Estimate	SE	p value
Anterior putamen			
*α*_1_	2.21	0.60	<0.001
*β*_1_	0.16	0.03	<0.001
*γ*_1_	0.02	0.01	<0.001
*δ*_1_	0.40	0.05	<0.001
Intercept	1.15	0.05	<0.001
Posterior putamen			
*α*_2_	1.80	1.46	0.218
*β*_2_	0.29	0.09	0.002
*γ*_2_	0.03	0.01	0.014
*δ*_2_	0.20	0.02	<0.001
Intercept	0.49	0.06	<0.001

Abbreviation: SE = standard error.

### 2.5 Estimation and Comparison of Function Values: Greater Damage at the Posterior Putamen vs Anterior Putamen at Symptom Onset

As shown in model (2), the estimated value of the curve in region 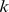 for a patient whose age at symptom onset was 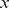 years, and for whom 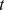 years had elapsed since this onset, is given by (suppressing patient and time indices):
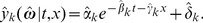
(5)


Assuming that 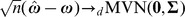, the delta method gives an approximate 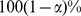 CI for the difference between estimated values in different responses [23], [24]:
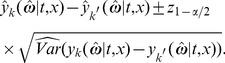
(6)


As an example, we compared the level of MP at symptom onset (i.e. 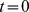) between the anterior and posterior regions of the putamen in the less affected side. The estimated intercepts for anterior and posterior putamen were 1.15 B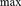/K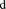 (95% CI 1.05–1.25) and 0.49 B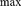/K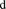 (95% CI 0.38–0.59), respectively. The difference between these two regions was statistically significant (0.67 B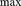/K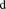, 95% CI 0.56–0.77). Clinical researchers concluded from these observations that the degree of damage to the dopamine system at symptom onset was significantly greater in the posterior putamen than in the anterior putamen.

### 2.6 Estimation and Comparison of Rates: Rates of Decline Differ between the Anterior and Posterior Putamen

The rates of decline of these measurements over the duration of symptoms characterize a major aspect of disease progression. They allow for year-to-year comparison of the decay rates for each tracer. The rate of decline of a given tracer in region 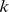 at time 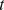 is given by (suppressing patient and time indexes):
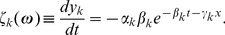
(7)


Thus, the rate depends on the parameters 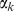, 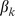, and 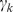. Assuming again that 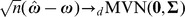, then the delta method gives an approximate 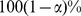 CI for the difference between rates [23], [24].

To illustrate, rates of decline in the measurements were compared for MP between the anterior and the posterior putamen in the less affected side, at each year since symptom onset (years 1 through 25). Although the coefficients of curvature 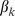 were not significantly different, the rates were found to be more rapid in the anterior putamen than in the posterior putamen: in the anterior putamen, rates were −0.054, −0.024, −0.011, and −0.005 B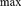/K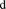 per year at 5, 10, 15, and 20 years since symptom onset, respectively, while the rates were generally slower in the posterior putamen, with −0.020, −0.005, −0.001, and 0.000 B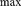/K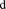 per year at 5, 10, 15, and 20 years since symptom onset, respectively. After Bonferroni adjustment for 25 multiple comparisons from years 1 through 25, there was evidence for a significantly sharper decline (i.e., statistically significant differences between rates) in the anterior putamen during the first 16 years of symptom duration followed by a more parallel decline (i.e., no statistically significant differences between rates) in the two regions during the later part of the disease (Figure 1). The difference in rates at different times could be explained by a delay or lag in the degree of damage due to the disease in the less affected side.

### 2.7 Estimation of Convergence, Divergence, or Parallelism: the Less Affected and the more Affected Sides Converge Over the Course of the Illness

Convergence, divergence, or parallelism of curves over the duration of symptoms was tested to compare the pattern of progression in different regions or sides. This was done by analyzing the difference in the extent of separation between the initial (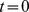) minus the final (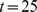) estimates of the corresponding curves. A significant positive difference in separation indicated convergence and a significant negative difference in separation indicated divergence.

For example, we compared the less affected to the more affected sides, by testing the convergence/divergence for MP measurements for each of the anterior and the posterior putamen regions separately. Convergence between the less and more affected sides was observed in both regions: The difference between the sides decreased significantly by 0.33 B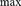/K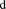 (95% CI 0.22–0.45) in the anterior putamen and by 0.14 B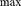/K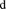 (95% CI 0.06–0.22) in the posterior putamen. Thus, over the course of the illness, the asymmetry in measurements between the less affected and more affected sides in both regions became less prominent, as the corresponding curves significantly converged.

### 2.8 Effect of Age at Symptom Onset: More Effective Compensatory Mechanisms in Younger Patients

**Figure 2:**
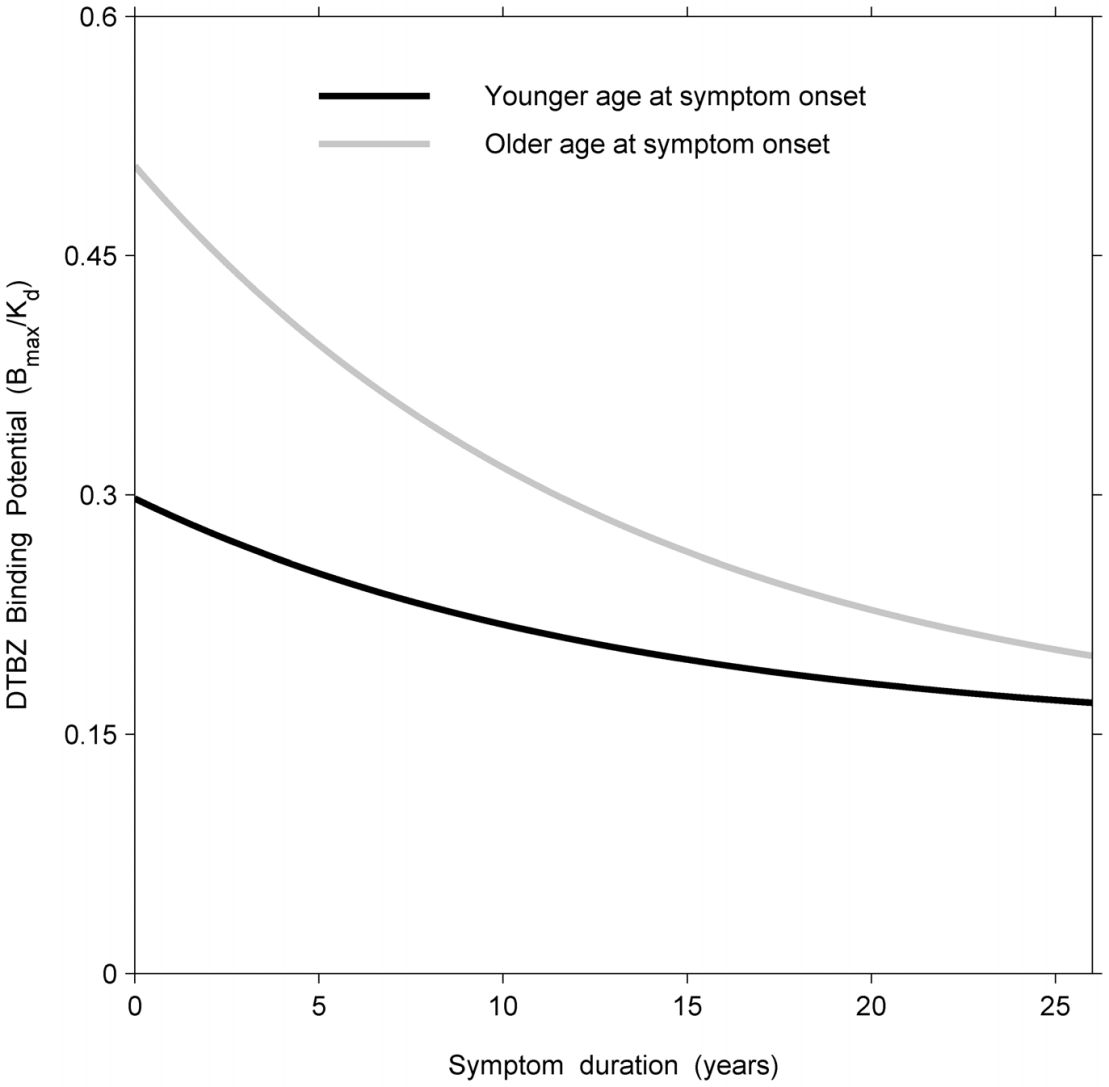
Longitudinal model fits of responses (DTBZ measurements) in the more affected side for the average of the three putaminal regions in the younger and older groups (35 and 70 years, respectively).

The trajectories of young versus old onset patients were estimated from the model using the complete set of data, and were then compared. We use as an example the measurements of DTBZ for the more affected side, averaged across the three putaminal regions. Young patients were 35 years of age at symptom onset (5th percentile by age) and older patients were taken to be 70 years of age at symptom onset (95th percentile by age). At the corresponding times of symptom onset (i.e., 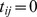), the values of DTBZ derived from the model were 0.30 (95% CI 0.21–0.38) and 0.51 (95% CI 0.40–0.61) for younger (i.e., 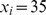) and for older (i.e., 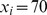) patients, respectively. Thus, younger patients had significantly lower values of DTBZ compared with older patients (average difference at time of symptom onset = −0.21, 95% CI −0.38– −0.04) (Figure 2). For the normal subjects, tracer measurements were modelled as a linear function of age at the time of visit with a random effect for the intercept (see model (8)). Relative to the fitted value for age-matched normal subjects, younger patients (i.e., 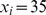) had lost 71% and older patients (i.e., 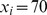) had lost 34% of DTBZ binding at symptom onset (i.e., when 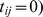. This finding suggests that younger patients lost considerably more dopamine nerve terminals before manifesting clinical evidence of PD, and suggests the presence of more effective compensatory mechanisms at a younger age.

### 2.9 Estimation of Preclinical and Presymptomatic Duration of Disease: Disease began Long before Symptoms

For our PD patients, the tracer-based preclinical and presymptomatic *duration* of disease was defined to be the time point prior to symptom onset beginning at the time at which tracer measurements for a patient first departed from those for a normal subject. Measurements in normal subjects were found to be linearly related to age: linear mixed effects regression was used to model longitudinal responses as a function of current age in our normal control subjects. We derived the corresponding normal control function for each of the three tracers. The mean value for a given response for normal control subject 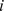 at visit 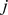 is given by
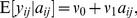
(8)where 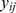 represents the tracer measurement for subject 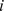 at the 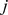
th visit, 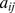 is the current age at visit 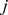, and 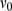, 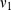 are the intercept and slope parameters, respectively.

To relate our exponential PD functions (model (2)), which were regressed on 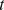, the duration of symptoms, to the control data, regressed on age, we expressed current age, 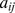, as the sum of the duration of symptoms, 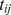, and age at symptom onset, 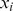, so the modelled measurements for normal subjects (model (8)) could also be expressed as functions of duration of symptoms:
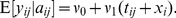
(9)


For a given age at symptom onset, 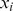, the intersection between the fitted line for normal subjects (model (9)) and fitted curve for PD patients (model (2)) yielded an extrapolated estimate of the preclinical duration of disease [25].

We first fixed the average age of symptom onset of the patients at 53 years, the observed mean age of symptom onset in our data. (Similar analyses could be carried out with any other observed values of age of symptom onset in the data). This limited the back-extrapolated analysis of the exponential curve to the one single remaining time variable in the model, viz. the duration in years prior to symptom onset. This time variable of duration was directly linked to the corresponding age variable for the normal controls by the simple relation: age = duration +53.

We then solved the exponential (patient) function and the linear (control) function simultaneously, and found their common point of intersection. The coordinates of this point estimate provided a value X(0) for the duration and a value Y(0) for the corresponding tracer level, at the time of the theoretical onset of the patients’ disease.

We next calculated a 90% confidence interval for the tracer level Y about Y(0), using the estimated standard error of the exponential curve at that duration to establish conservative bounds. We then identified the two points on the exponential curve that corresponded to the upper and to the lower limits of the confidence interval for Y. From both points, perpendiculars were dropped onto the X-axis, and the resulting values X(1) and X(2) were used as the corresponding 90% “fiducial limits”, likely to contain the true value of the pre-symptomatic duration of disease onset.

We used the R function uniroot, a one dimensional root finding algorithm, to solve for the estimated preclinical duration [26], [27]. We used the unique root *prior* to symptom onset for a given age at symptom onset.

As an application, the measurements of DTBZ were modelled for the more affected and the less affected sides, averaged across the three putaminal regions. Curves for the more affected and less affected sides, for the average age at symptom onset of 53 years, were extrapolated backward from duration 0 (i.e., time of symptom onset) to their points of intersection with the corresponding tracer-matched regression lines for the normal control subjects. Using methods of inverse regression, the preclinical durations of disease for both sides were derived [25]. The presymptomatic period for the more affected side was 17 years (90% fiducial limits 2–24) and for the less affected side was 8 years (90% fiducial limits 4–11). We regard these estimates with some caution due to the combined uncertainty in extrapolation and inverse regression. However, we present these tentative results due to their highly interesting implications.

### 2.10 Comparisons of Standardized Responses among Tracers: Differences in Tracer Information Over Time Show Diminishing Effects of Early Compensation

Thus far, we have described methods for comparing parameter estimates, estimated functional values, comparisons of rates of decay, tests of parallelism, effects of age of symptom onset, and derivation of age at disease onset. Such comparisons are feasible when the multivariate responses are on a commensurate scale. When comparing different tracers, however, such as FD and DTBZ, the corresponding responses are measured on different scales. To carry out comparisons between tracers, we therefore needed to standardize their measurements relative to the corresponding values of the age-matched normal controls. In the control data, however, tracer values decline at different rates with normal aging. We therefore standardized our tracer measurements by dividing the PD measurements by the corresponding normal expected values at the same age, derived from the linear regressions for the control subjects. Thus the disease process could be compared across different tracers using our model and methods as described above.

**Figure 3:**
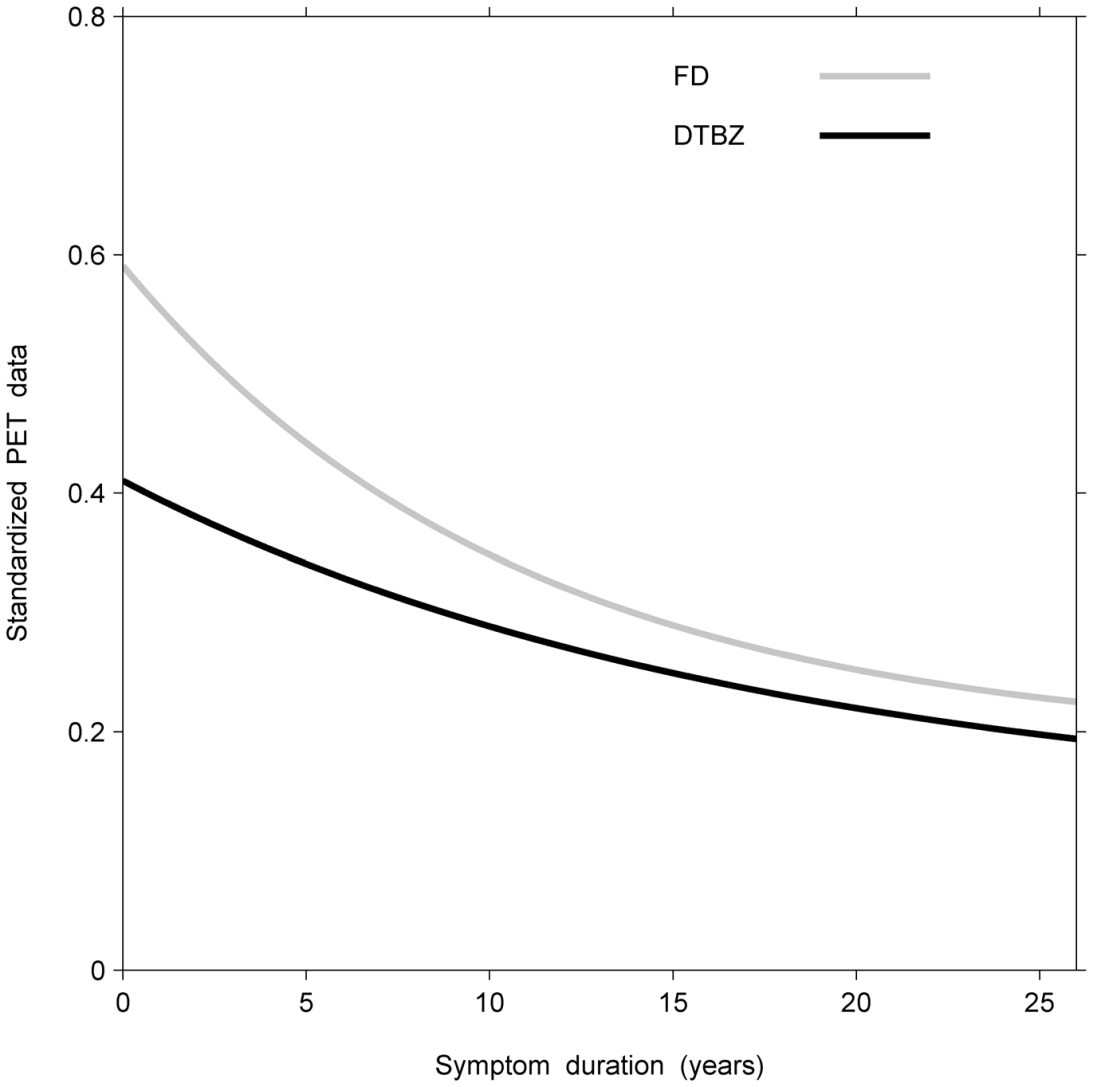
Longitudinal, bivariate model fits of standardized responses (FD and DTBZ measurements) in the more affected side averaged across the three putaminal regions. This suggests that the effects of early compensation dissipate over time (see text).

As an illustration, the standardized measurements of FD and DTBZ were modelled as above for the more affected side averaged across the three putaminal regions (Figure 3). Thus, at duration 0, the proportional loss in FD relative to its normal controls was significantly less than that of DTBZ (a difference of 0.18, 95% CI 0.11–0.25). This suggested that despite overall nerve terminal loss, differential compensatory changes occur, reflected in differences between the tracers at symptom onset. Moreover, the standardized DTBZ curve remained below the standardized FD curve throughout the entire duration of symptoms. Significant curvature was present for FD (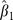 = 0.09, 95% CI 0.03–0.15) and DTBZ (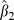 = 0.06, 95% CI 0.01–0.10), but did not differ significantly between the two tracers. The rates of decline were significantly different between the two tracers from 3 to 10 years since symptom onset, after Bonferroni adjustment for multiple yearly comparisons. This suggests the effects of early compensation which dissipate over time.

### 2.11 Comparison of Fitted Models: Longitudinal vs. Cross-sectional Analysis: Potential biases and Information Gain

**Figure 4:**
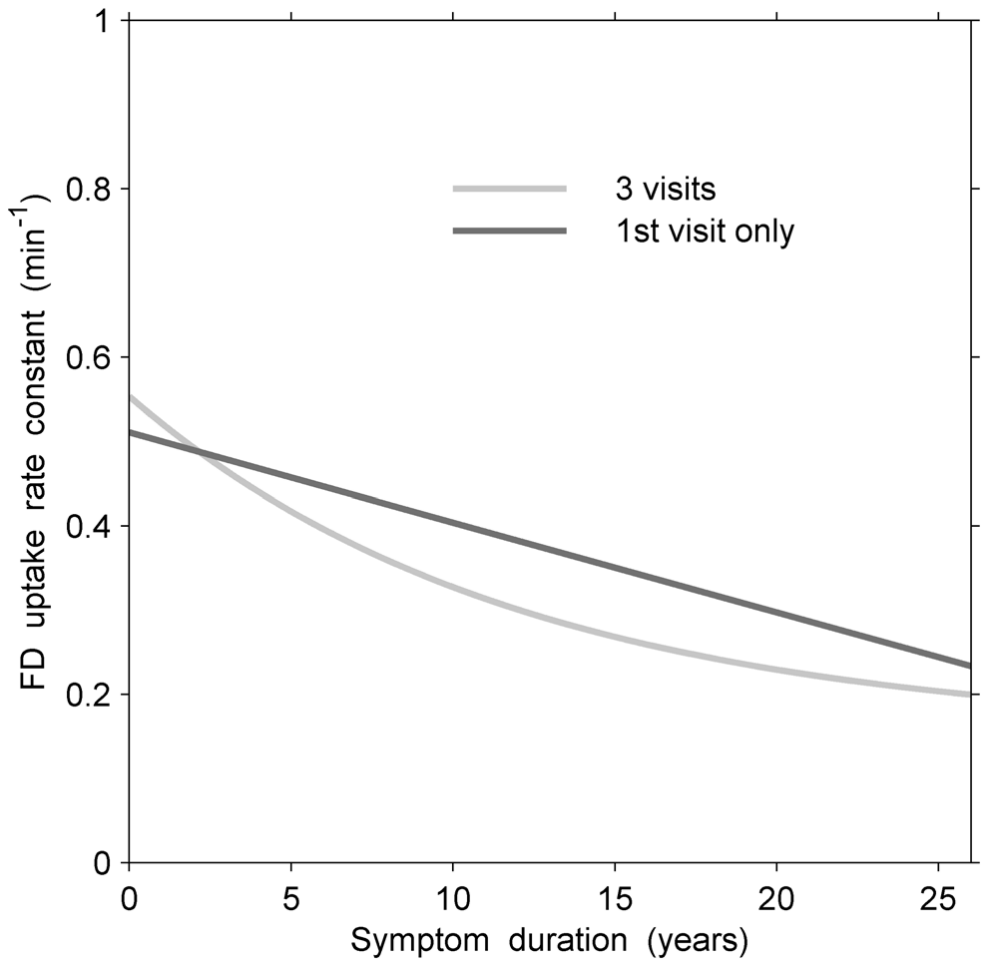
Longitudinal (all 3 visits) and cross-sectional (1st visits only) model fits of responses (FD measurements) in the more affected side averaged across the three putaminal regions for patients with an average age at symptom onset of 53 years. There was little suggestion of bias due to selective loss of patients in the longitudinal approach. For example, the rate of decline in FD uptake at 15 years since symptom onset was about −0.0095 min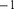 per year from the longitudinal curve based on 3 visits and −0.0107 min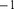 per year from the cross-sectional curve based on those patients who only provided a single visit.

To examine potential biases in our PD longitudinal vs. cross-sectional data and to assess the gain in efficiency of the information from our longitudinal data, we compared estimated non-linear models for FD in the more affected side averaged across the three putaminal regions and fitted to patients with data from all 3 visits vs. those with data from 1 visit only (Figure 4). The comparison clearly indicated that both curves closely agree in their description of the course of general decline of the disease, suggesting that no important potential bias was present due to loss of follow-up. While the longitudinal analysis yielded a significant curvature (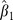 = 0.08, 95% CI 0.00–0.17), the cross-sectional model was not sufficiently sensitive to detect curvature (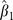 = 0.02, 95% CI −0.06–0.10). Compared to modelling data cross-sectionally based on a single visit, the relative efficiency for the estimate of the curvature, 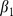, from data obtained longitudinally from all 3 visits, offered a 20% improvement in precision. In other cases, such as models for DTBZ in the less affected side of the anterior putamen, the corresponding relative efficiency reached a 5-fold improvement in precision for 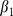.

Similarly, the longitudinal slopes of the regression lines for the normal controls agreed closely with those derived from subjects who provided only a single measurement, suggesting the absence of any significant, longitudinal bias and supporting “non-informative missingness”.

## Discussion

We derived non-linear random effects models for multivariate responses of PET data measured longitudinally in PD patients. In order to study the temporal and the spatial dynamics of PD progression, serial PET measurements were collected bilaterally from striatal subregions of PD patients using three different tracers. Previous limited PET studies of progression in PD have indicated some non-linear trends, but have not fully taken into account the multivariate and longitudinal aspects of their data [11]. Our work has applied and enlarged on the recently developed methodology of non-linear random effects models for multivariate responses [10]. This permitted us to analyze in greater detail the nature of the progression and of the pathogenesis of PD, as well as its preclinical aspects.

Our clinical results [28]–[30] have provided considerable insights into the evolution of the disease. In particular, we concluded that the degree of damage to the dopamine system at symptom onset was greater in the posterior putamen than in the anterior putamen (Section 2.5). This conclusion was based, inter alia, on the difference in MP intercepts between the anterior and posterior regions in the less affected side of the brain. This difference was supported also by comparison of intercepts of other tracers.

From the comparison of asymptotes, we concluded that MP levels remained functionally different between the anterior and posterior regions of the putamen (Section 2.4).

In addition, while significant curvatures were detected within each of these anterior and posterior putamen regions, the curvatures did not differ significantly between these regions (Section 2.4). In fact, the overall rates of decline were initially greater in the anterior putamen, but became similar in both regions as the disease progressed (Section 2.6).

Convergence in MP levels between the more and less affected sides of both the anterior and posterior putamen indicated that over the course of the illness, differences between the sides tend to disappear (Section 2.7).

The comparison of trajectories of DTBZ for the more affected side averaged across the three putaminal regions indicated that patients with younger symptom onset had lost more dopamine nerve terminals prior to the onset of their symptoms than had older onset patients (Section 2.8), thus suggesting that younger patients had more effective compensatory mechanisms at their disposal.

A comparison of DTBZ and FD measurements for the anterior putamen regions on the more affected side showed that despite overall nerve terminal loss, differential compensatory mechanisms occur between these two tracers at symptom onset. This results in significant early differences in the degree of loss corresponding to normal controls in each tracer (Section 2.10).

Cautionary results were extended to the pre-clinical phase of the disease. They provided a valuable estimate of the latency period between disease onset and clinical onset (Section 2.9).

The conclusions derived from the application of our model provide important biological insights. The combination of differential involvement of striatal subregions at *disease onset* with similar rates of decline over time between subregions can be taken to imply that the factor(s) that contribute to disease initiation (and are regionally selective) may be different from those that underlie disease progression (which appear to be more uniform). This has important implications for the understanding of PD and consequently for the design of disease-modifying therapies. The observation that earlier age of *disease onset* is associated with a greater degree of denervation prior to *symptom onset* implies that younger subjects have more effective compensatory mechanisms (and are hence able to tolerate greater degrees of denervation). In summary, the application of this statistical model has allowed us to understand the neurochemical underpinnings of the PD pathogenesis *in vivo*. Our methodology can be applied to study many other chronic progressive diseases.
